# A Head-to-Head Comparison of Three Self-Help Techniques to Reduce Body-Focused Repetitive Behaviors

**DOI:** 10.1177/01454455211010707

**Published:** 2021-04-21

**Authors:** Steffen Moritz, Danielle Penney, Kaser Ahmed, Stella Schmotz

**Affiliations:** 1 University Medical Center Hamburg-Eppendorf, Hamburg, Germany; 2 Centre Intégré Universitaire de Santé et de Services Sociaux de l’Ouest-de-l’Île-de-Montréal, Douglas Mental Health University Institute, Canada

**Keywords:** body-focused repetitive behaviors, trichotillomania, skin picking, nail biting, habit reversal training

## Abstract

Body-focused repetitive behaviors (BFRBs) include skin picking, trichotillomania, nail biting and cavitadaxia/lip-cheek biting, among other behaviors. For the first time, we compared three different self-help techniques aimed at reducing BFRBs. We explored the acceptance and preliminary efficacy of the approaches and whether the techniques exerted differential effects depending on BFRB-type.

A total of 113 participants with at least one BFRB were randomly allocated to either habit reversal training (HRT; active elements: awareness and competing response training), decoupling (DC) or decoupling in sensu (DC-is). Reassessment was conducted 4 weeks later. The Generic Body-Focused Repetitive Behavior Scale (GBS) served as the primary outcome. The completion rate was best for DC-is (68.6%) as compared to HRT (57.1%) and DC (53.5%). A total of 34.8% of completers in the DC group showed an improvement of at least 35% on the GBS compared to 10.0% in the HRT and 23.3% in the DC-is group. In accordance with previous work, moderator analyses showed that improvement under DC is best for non-skin-pickers. A dose-effect relationship emerged, particularly for HRT. Subjective appraisal ratings were more favorable for DC-is and HRT than for DC. With respect to completion rate, subjective appraisal and symptom improvement, DC-is yielded consistently satisfactory results, whereas HRT showed good subjective but rather poor objective improvement. Those who performed DC, especially non-skin-pickers, showed good improvement but overall completion and subjective efficacy were low. Future studies should investigate whether the three techniques exert add-on effects when combined and whether demonstration via new media (e.g., video) will augment comprehensibility and thus efficacy of the techniques.

## Introduction

Body-focused repetitive behavior (BFRB) is a heterogeneous diagnostic entity which most often manifests as skin picking or dermatillomania (i.e., the repetitive scratching, biting, and picking of the skin), dermatodaxia/”wolf biting” (Scott & Scott, 1997) (i.e., the gnawing and biting of the skin adjacent to the fingers), trichotillomania (the pulling out of body hair), nail biting, and cavitadaxia/lip/lip-cheek biting. In the latest edition of the Diagnostic and Statistical Manual of Mental Disorders (DSM-5; APA, 2013), BFRB is subsumed under the chapter “obsessive-compulsive and related disorders” (Van Ameringen et al., 2014). Prevalence rates are inconsistent across studies depending on mode of assessment (self-report vs. expert rating) and rigor of criteria (e.g., including or excluding noticeable damage) (Hayes et al., 2009). To illustrate, one study found forms of lip/cheek biting in approximately two out of five people (Teng et al., 2002), while other studies reported prevalence rates of 2 or 3 out of 100 people (Shulman, 2005; Shulman et al., 2004).

While some reviews recommend selective serotonin reuptake inhibitors or N-acetyl cysteine for the treatment of BFRBs (Lochner et al., 2017), to date no medication has been approved by the Food and Drug Administration (FDA) for BFRBs. Habit reversal training (HRT) (Azrin & Nunn, 1973) is widely considered the treatment of choice for BFRB according to meta-analyses (Bate et al., 2011) and systematic reviews (Lee et al., 2019; Lochner et al., 2017); HRT involves a series of techniques including an observational period to elucidate triggers/situations that commonly elicit the behavior (i.e., awareness training) as well as progressive muscle relaxation. Its main technique is competing response training which instructs the participant to perform a static “freezing” alternative behavior (e.g., clenching of the fist for some time, sitting on one’s hands). Habit reversal training is usually conducted by a therapist.

More recently, our group has developed two additional self-help treatments: decoupling (DC) (Moritz et al., 2011; Moritz & Rufer, 2011) and decoupling in sensu (DC-is) (Moritz, Rufer, et al., 2020), which we consider variants of HRT. In decoupling, the participant performs a movement resembling the typical approach sequence of the dysfunctional behavior. Shortly before the (un)desired behavioral target is reached and the behavior completed (e.g., tearing out a hair in trichotillomania), the participant redirects the movement instead targeting another location on the body or a particular point in the room using an accelerated movement of the hand (for trichotillomania, skin picking, or nailing biting) or part of the mouth (cavitadaxia). Decoupling aims to divert the behavior and to create an irritation once the often automatic BFRB is initiated so that the dysfunctional behavior can be detected early enough to allow the individual to intervene. Similar to HRT, in decoupling the individual is first advised to observe the undesired behavior without interfering. This enables the identification of typical motor patterns as well as triggers and instances when the behavior is prevalent. Decoupling has been successfully tested for nail biting (Moritz et al., 2011) and trichotillomania (Moritz & Rufer, 2011; Weidt et al., 2015). A case report with favorable results has been published for cavitadaxia (Moritz, Müller, et al., 2020). In their systematic review, Lee et al. (2019) conclude that “Throughout the review, we found evidence of benefit for “variants” of HRT, for example “movement decoupling” (p. 13).

Regarding decoupling in sensu, the individual is instructed to close his or her eyes and imagine different motor sequences that typically precede the BFRB (e.g., imagining the hand close to the mouth and his or her teeth about to bite or gnaw the skin of the adjacent fingernail). Shortly before the completion of the BFRB (e.g., biting the skin), the imagined movement is interrupted by an actual movement; the hand (or other body part) that had been previously just imagined should be clenched into a fist and moved quickly downwards with the fingers spread wide. Thus, the imagined sequence is terminated by a behavioral counter-response. Compared to the other methods, empirical evidence in favor of decoupling in sensu is currently weak. A single case report exists wherein a 50-year-old male successfully overcame his perionychophagia and skin picking using this technique (Moritz, Rufer, et al., 2020).

To the best of our knowledge only one study performed a direct comparison of two of the techniques. A total of 70 individuals with skin picking behaviors were randomly allocated to either HRT or DC, which were delivered as self-help interventions. Half of participants in the HRT group reported symptom decline relative to one third in the DC group (Moritz et al., 2012). The specific aims of the present study were to 1. conduct a head-to-head comparison of the three treatment methods (HRT, DC, DC-is), 2. use a transdiagnostic approach to examine whether participants with different BFRBs benefit from the different techniques, and 3. implement a generic form of the Skin Picking Scale-Revised (German version, Mehrmann et al., 2017; Snorrason, Ricketts, et al., 2012) devised by our group to allow for the assessment of different BFRBs.

## Methods

### Sample

The study was conducted online in the framework of a randomized controlled trial without stratification. Assessments were carried out using Questback/UniPark^®^. The study was advertised on Facebook forums and on our website (www.uke.de/decoupling) as an unguided treatment study for people with BFRBs (its different manifestations were described in detail to potential participants). Individuals were allocated to either HRT (which while usually conducted by a therapist, was adapted for delivery as a self-help intervention), decoupling or decoupling in sensu (allocation: 1:1:1). Participation was anonymous and individuals were instructed on how to create anonymous email addresses. Inclusion criteria were to be at least 16 years of age with at least one self-reported BFRB. Concurrent treatments (e.g., medication, other self-help intervention, psychotherapy) were tolerated. The final analyses included data from 113 participants. The trial was registered with the German Clinical Trials Register (DRKS00022560).

### Invitation, Baseline, and Post-assessment (Self-Report)

In keeping with the guidelines of the European General Data Protection Regulation (GDPR), the online survey did not store IP addresses. Ethical approval was obtained from the local ethics committee for psychologists at the University Hospital of Hamburg-Eppendorf (Germany, LPEK-0179). As an incentive, participants received all three self-help manuals upon completion of the post-assessment. Following a brief introduction to the study, electronic informed consent was requested as a mandatory precondition for participation. Subsequent to questions on age and gender, we asked whether individuals had ever (i.e., lifetime prevalence) suffered from skin picking, nail biting, tearing out the hair, biting the skin (or mouth) or other BFRBs resulting in either injuries, visible consequences (e.g., gnawed fingernails) or disabilities in social, occupational or other important areas of life (participants could endorse multiple options). The same questions were then repeated but the time frame was confined to the last 2 weeks. After that, we asked for participants’ medical history (e.g., number of psychotherapy consultations; prior participation in studies assessing psychological/psychotherapeutic treatments; any psychiatric diagnoses; current pharmacological treatment). Two psychological scales were administered (see below). At the end of the baseline assessment, we asked whether all questions had been answered truthfully and then requested an anonymous email address. Participants were then automatically randomized based on the date of participation to one of the three conditions and immediately received an automatic “trigger email” with the manual attached. Thus, concealed allocation was ensured. Four weeks following initial engagement in the study, all participants were automatically invited to take part in the post-assessment and received up to three reminders to complete it. Participants were asked to re-enter their email address and were then prompted to complete the same scales as before, this time considering a 1-week time frame. We asked participants to what degree they had used the manual, which was measured on a 7-point scale (not read at all; partially read but never used; used technique once in the last 6 weeks; used technique once weekly; used technique multiple times weekly; used technique on a daily basis; used technique several times daily). For those who indicated that they had at least started to read the manual, we posed further questions related to subjective quality, comprehensibility, satisfaction, subjective efficacy and frequency of administration (see below). We also asked participants what they had liked or disliked about the study or the intervention technique (free text, optional). We also asked whether prior responses were made truthfully (yes, no). Before terminating, participants had the option to download all manuals.

### Primary Outcome

The Generic Body-Focused Repetitive Behavior Scale (GBS) represented the primary outcome. The scale relies on the items of the Skin Picking Scale (SPS) (Keuthen et al., 2001) in its revised 8-item form (SPS-R; Snorrason, Ricketts et al., 2012). The SPS-R has acceptable psychometric properties according to its authors and has been verified for the German version which includes good internal reliability for its modified German version (alpha = . 81 for its total score) as well as high convergent validity with the Skin Picking Impact Scale (Mehrmann et al., 2017). The scale was slightly reformulated to capture different forms of BFRBs. Analogous to the Yale-Brown Obsessive-Compulsive Scale (Y-BOCS) (Goodman et al., 1989), which asks individuals with obsessive-compulsive disorder to provide pooled estimates for different forms of obsessions and compulsions, participants were instructed to provide a joint rating in the event that they suffered from different BFRBs. We provided an example of an individual who gnawed his or her nails for approximately 1 hour and would engage in skin picking for 3 hours daily. In this case, the correct estimate would be 4 hours. If the urge to execute one specific BFRB was minor but strong for another, the maximum urge (strong) should be entered (same for other items). For fluctuations, the mean score should be estimated. Every item had to be scored on a 5-point Likert scale ranging from 0 to 4. Similar to the German adaptation of the SPS-R and in keeping with the Massachusetts General Hospital (MGH) Hairpulling Scale (Keuthen et al., 1995), control over the behavior was differentiated into 1. control over the urge, and 2. behavior; the sum score contains the mean of these two ratings. The time frame was set to the last 2 weeks (baseline) or the last week (post-assessment). Items included: 1. Frequency of the urge to perform BFRB (*from* 0 = no urge *to* 4 = constant urge (>8 hours per day)), 2. Intensity of the urge to perform BFRB (*from* 0 = no urge *to* 4 = extremely strong urge), 3. Control over the urge (*from* 0 = no urge or could always control urge *to* 4 = urge could not or only hardly be resisted), 4. Time spent performing BFRB (*from* 0 = no behavior *to* 4 = behavior performed almost constantly (> 8 hours per day)), 5. Control over the BFRB (*from* 0 = full control: could always resist or terminate *to* 4 = no control, can never stop); 6. Emotional distress/suffering because of BFRB (*from* 0 = not at all *to* 4 = very high emotional distress, self-injurious behavior stressed me a lot), 7. Impairment in social and occupational functioning (*from* 0 = no impairment *to* 4 = extreme impairment), 8. Avoidance (*from* 0 = no avoidance *to* 4 = almost constant avoidance), 9. Acute somatic consequences (0 = no injuries *to* 4 = very severe injuries). In keeping with the factorial structure of the SPS-R obtained in the original study (Snorrason, Ólafsson, et al., 2012), we calculated two subscales as well as a total score (the order of items deviates from the original SPS-R). The symptom severity subscale was composed of the sum of items 1, 2, 4, and the mean rating of items 3 and 5. The impairment subscale was composed using the sum of items 6 to 9.

### Secondary Outcome

The global item on the WHO Quality of Life (WHOQOL-BREF) (Skevington et al., 2004) was used as an index of quality of life in the last 2 weeks.

### Reliability of Measures

For the present study, test-retest reliability of the GBS was satisfactory for the total score (*r* = 0.760, *p* < .001) and the impairment subscale (*r* = 0.854, *p* < .001), but was low for the severity subscale (*r* = 0.540, *p* < .001). Cronbach’s alpha was satisfactory for all scales: severity (α = .748), total score (α = .787) and impairment (α = .813). The test-retest reliability of the GBS was *r* = 0.77 (*p* < .001) in an independent study, where participants did not receive an active intervention during a 6-week period (*n* = 28). In an independent (unpublished) study we validated the GBS against the Repetitive Body Focused Behavior Scale (RBFBS, self-report) (Selles et al., 2018), adapted for adults, in 21 patients with satisfactory results (*r* = 0.51, *p* = .019).

### Subjective Appraisal and Benefit

Participants who had read the manual were asked to complete the Patient Satisfaction Questionnaire (German acronym ZUF-8; Schmidt et al., 1989), adapted for online interventions. The ZUF-8 assesses subjective appraisal of the technique (e.g., quality, satisfaction, subjective efficacy, intention to use the application in the future). Table 4 and 5 show the results of the ZUF-8 and additional questions from the Subjective Efficacy Scale (e.g., Moritz et al., 2019) regarding the treatment.

### Intervention

The self-help manuals were emailed as pdf-files (free download at www.uke.de/decoupling).

The HRT technique was outlined in an eight-page PDF file. The protocol taught two main components of HRT. First, the phenomenology of different BFRBs was described as well as possible somatic and psychological consequences. The technique consists of two steps: 1. prior to the application of the exercises, the participant was asked to identify situations that trigger BFRB and the times when BFRBs were most prevalent (awareness training). Second, participants were instructed to perform antagonistic (static) behaviors for 1 to 3 minutes once an urge to skin-pick was noticed (competing response training, CRT) or as a means to stop an ongoing BFRB. Several examples were provided and illustrated with photos.

The DC technique was outlined in a 12-page PDF file. The introduction was essentially identical to the HRT manual and was followed by the theoretical rationale of DC. Participants were instructed on how to shape/deviate their dysfunctional behavior into a similar but benign behavior by means of decoupling core behavioral elements. Movements that resembled the initiating phase of the previous dysfunctional behavior should be targeted first. In close proximity to the prior behavioral target (e.g., nails) the movement should be deviated and target either another location on the body or a certain point in the room with *an accelerated movement*. Instructions were illustrated by a sequence of photos. Decoupling can be practiced in both symptomatic and symptom-free intervals.

The DC in sensu technique was outlined in an eight-page PDF file. Again, the introduction was essentially identical to the other two manuals. Subsequently, the theoretical rationale of DC in sensu was explained, which is similar to DC. Unlike in DC, which is executed at an entirely behavioral level, movements that resemble the previous dysfunctional behavior should be *imagined*. Shortly before the completion of the BFRB (e.g., biting the skin), the imagined movement is interrupted by an actual movement. The hand, in the case of nail biting, that was previously imagined should in reality be clenched into a fist and moved quickly down with the fingers spread wide. Thus, the imagined sequence is terminated by a behavioral counter-response. The revised decoupling protocol is hoped to allow for greater generalization than conventional decoupling (Moritz, Rufer, et al., 2020).

## Results

A total of 144 participants started the baseline survey, 14 terminated their participation prematurely or endorsed in the final item that they had not answered truthfully. Six participants had an age below 16 years. Of the remaining 124 individuals data from 11 participants were not retained (blind to results) for the following reasons: a score of 0 on the GBS (*n* = 6), investigator error leading to failure to contact for post-assessment (*n* = 3), participant had obtained all manuals due to repeated registration (*n* = 1) and untrustworthy/inconclusive entries (participant entered “7 × 7” when asked for age, *n* = 1). All remaining participants (*N* = 113, intention-to-treat sample) endorsed that they had answered all questions truthfully. There were no reported instances of schizophrenia or a history of severe substance use.

Demographic characteristics are displayed in Table 1. The majority of participants were female and in their late 20’s/early 30’s. Most participants had no further psychiatric diagnoses (based on self-report). Depression was reported in approximately one fourth of the cases; obsessive-compulsive disorder was rare (5.7%–11.6% of the cases). Nail biting and trichotillomania were most prevalent in the sample followed by skin picking and cavitadaxia. Participants had an average total of 1.76 BFRBs (*SD* = 0.88; single BFRB = 46.9%; two BFRBs = 36.3%; three BFRBs = 10.6%; four BFRBs = 6.2%). Samples did not differ regarding current medication of mood-related problems nor with respect to the number of prior psychotherapies.

**Table 1.** Baseline Characteristics of the Sample. Means, Standard Deviations and Frequencies.

	HRT (*n* = 35)	DC (*n* = 43)	DC-is (*n* = 35)	Statistics
Demographic and clinical characteristics				
Age in years	34.26 (11.00)	29.79 (10.63)	35.03 (13.37)	*F* (2, 112) = 2.35, *p* = .100
Gender (female/male/undetermined)	22/13/0	37/5/1	25/10/0	χ^2^ (4) = 8.445, *p* = .077
Medication (antidepressant/mood stabilizer)	30/5	38/5	31/4	χ^2^ (2) = 0.169, *p* = .919
Prior psychotherapeutic treatment	0.66 (1.14)	0.61 (1.03)	0.60 (1.09)	*F* (2, 112) = 0.31, *p* = .970
Global BFRB Scale (GBS)				
Total score	14.86 (4.81)	14.92 (4.47)	15.20 (4.77)	*F* (2, 112) = 0.05, *p* = .947
Severity	8.46 (2.83)	9.17 (2.62)	9.14 (2.24)	*F* (2, 112) = 0.90, *p* = .410
Impairment	6.40 (4.45)	5.74 (2.68)	6.06 (3.29)	*F* (2, 112) = 0.42, *p* = .655
Quality of life				
WHOQOL BREF Global item	3.43 (0.88)	3.51 (0.77)	3.69 (0.80)	*F* (2, 112) = 0.91, *p* = .403
Lifetime prevalence of body-focused repetitive disorders				
Skin picking	34.3%	41.9%	54.3%	χ^2^ (2) = 2.914, *p* = .233
Nail biting	48.6%	46.5%	62.9%	χ^2^ (2) = 2.335, *p* = .311
Trichotillomania	48.6%	62.8%	51.4%	χ^2^ (2) = 1.818, *p* = .403
Cavitadaxia	22.9%	18.6%	28.6%	χ^2^ (2) = 1.083, *p* = .582
Lifetime prevalence of psychiatric disorders				
No psychiatric diagnosis	65.7%	58.1%	60.0%	χ^2^ (2) = 0.490, *p* = .783
Depression	25.7%	25.6%	28.6%	χ^2^ (2) = 0.107, *p* = .948
Obsessive compulsive disorder	8.6%	11.6%	5.7%	χ^2^ (2) = 0.841, *p* = .657
Severe substance use	0%	0%	0%	—

*Note*. DC = decoupling; DC-is = decoupling in sensu; HRT = habit reversal training.

### Adherence and Efficacy

Completion rate was 68.6% in the DC-is condition, 57.1% in the HRT condition and 53.5% in the DC condition (total group: 67/113, 59.3%), χ^2^ (2) = 1.92, *p* = .382.

Most participants disclosed through self-report that they had at least read the manual with no differences across conditions (HRT: 95.0%, DC: 95.7%, DC-is: 91.7%; χ^2^ (2) = 0.380, *p* = .827). A total of 70.0% of the HRT group performed the exercises, compared to 82.6% in the DC group and 74.6% in the DC-is group, χ^2^ (2) = 1.182, *p* = .579.

Table 2 shows changes in symptom severity and quality of life across time using complete cases and intention-to-treat analyses (expectation maximization). At a large effect size, overall improvement, irrespective of condition, was found for the GBS total score and severity score irrespective of condition. A medium-to-large effect was observed for GBS impairment and quality of life. No significant interaction effects were observed. Greater improvement in quality of life was observed at a medium effect in the HRT and DC conditions, with stable scores for DC-is.

**Table 2.** Changes in Symptom Severity and Quality of Life across Time (ITT Sample, HRT, *n* = 35, DC, *n* = 43, DC-is, *n* = 35).

	HRT		DC		DC in sensu		Complete cases	Per protocol; read, exercises not performed	Per protocol; exercises performed	Intention to treat; expectation maximazation
	Pre	Post	Pre	Post	Pre	Post		T = Time; I = Interaction		
GBS total score	15.52 (4.93)	13.50 (4.57)	14.93 (4.49)	12.61 (5.54)	14.62 (5.14)	12.85 (6.48)	T: *F* (1, 64) = 20.14, *p* < .001, η_p_^2^ = .239	T: *F* (1, 60) = 21.21, *p* < .001, η_p_^2^ = .216	T: *F* (1, 47) = 22.70, *p* < .001, η_p_^2^ = .326	T: *F* (1, 110) = 53.23, *p* < .001, η_p_^2^ = .326
							I: *F* (2, 64) = 0.13, *p* = .877, η_p_^2^ = .004	I: *F* (2, 60) = 0.16, *p* = .852, η_p_^2^ = .005	I: *F* (2, 47) = 0.08, *p* = .920, η_p_^2^ = .004	I: *F* (2, 110) = 0.57, *p* = .567, η_p_^2^ = .010
GBS severity	9.17 (2.81)	7.70 (2.31)	9.11 (2.63)	7.83 (2.98)	9.04 (2.46)	7.60 (3.07)	T: *F* (1, 64) = 18.91, *p* < .001, η_p_^2^ = .228	T: *F* (1, 60) = 19.69, *p* < .001, η_p_^2^ = .247	T: *F* (1, 47) = 18.74, *p* < .001, η_p_^2^ = .285	T: *F* (1, 110) = 37.95, *p* < .001, η_p_^2^ = .256
							I: *F* (2, 64) = 0.03, *p* = .967, η_p_^2^ = .001	I: *F* (2, 60) = 0.35, *p* = .966, η_p_^2^ = .001	I: *F* (2, 47) = 0.36, *p* = .702, η_p_^2^ = .015	I: *F* (2, 110) = 0.34, *p* = .714, η_p_^2^ = .006
GBS impairment	6.35 (3.42)	5.80 (3.24)	5.83 (2.82)	4.78 (2.92)	5.58 (3.49)	5.25 (3.97)	T: *F* (1, 64) = 8.48, *p* = .005, η_p_^2^ = .117	T: *F* (1, 60) = 8.95, *p* = .004, η_p_^2^ = .130	T: *F* (1, 47) = 10.59, *p* = .002, η_p_^2^ = .184	T: *F* (1, 110) = 29.46, *p* < .001, η_p_^2^ = .211
							I: *F* (2, 64) = 0.95, *p* = .381, η_p_^2^ = .029	I: *F* (2, 60) = 0.88, *p* = .422, η_p_^2^ = .028	I: *F* (2, 47) = 0.11, *p* = .892, η_p_^2^ = .005	I: *F* (2, 110) = 0.90, *p* = .411, η_p_^2^ = .016
Quality of life global	3.45 (1.00)	3.85 (0.74)	3.43 (0.59)	3.65 (0.57)	3.67 (0.76)	3.67 (0.76)	T: *F* (1, 64) = 7.95, *p* = .006, η_p_^2^ = .110	T: *F* (1, 60) = 7.81, *p* = .007, η_p_^2^ = .115	T: *F* (1, 47) = 2.91, *p* = .095, η_p_^2^ = .058	T: *F* (1, 110) = 16.15, *p* < .001, η_p_^2^ = .142
							I: *F* (2, 64) = 2.49, *p* = .091, η_p_^2^ = .072	I: *F* (2, 60) = 2.43, *p* = .097, η_p_^2^ = .075	I: *F* (2, 47) = 0.43, *p* = .429, η_p_^2^ = .035	I: *F* (2, 110) = 6.37, *p* = .002, η_p_^2^ = .104

*Note*. DC = decoupling; DC-is = decoupling in sensu; HRT = habit reversal training; ITT sample = intention-to-treat: all cases considered, missing values imputed; Per protocol = complete data and participants had at least read the manual.

When using a 35% threshold criteria for improvement on the GBS, the difference between DC and HRT achieved a statistical trend for three out of four comparisons (i.e., non-significant finding; see Table 3). If non-completers were considered as non-responders approximately every fifth participant in the DC group showed symptom improvement in contrast to less than every 10th in the HRT condition.

**Table 3.** Treatment Response (35% Decline on the Global BFRB Scale) for Completer and Intention-to-Treat Sample (Non-Completion was Counted as Not Improved).

Item	HRT (*n* = 35) (%)	DC (*n* = 43) (%)	DC-is (*n* = 35) (%)	Statistics
Complete cases	10.0	34.8	23.3	χ^2^ (2) = 4.50, *p* = .106 (DC > HRT: .055)
Intention to treat	5.7	18.6	12.4	χ^2^ (2) = 3.00, *p* = .223 (DC > HRT: .090)

*Note*. DC = decoupling; DC-is = decoupling in sensu; HRT = habit reversal training.

### Dose-Effect Relationship

There was a significant dose-effect relationship for frequency of practice and reduction of GBS scores (*r* = 0.410, *p* = .001), especially for HRT (*r* = 0.567, *p* = .009) but at least medium effect sizes were observed for DC-is (*r* = 0.360, *p* = .084) and DC (*r* = 0.343, *p* = .109).

### Subjective Efficacy and Feasibility

Tables 4 and 5 demonstrate that acceptance and subjective efficacy were satisfactory-to-good across all conditions, with DC receiving mainly the lowest and DC-is and HRT receiving the highest ratings. Improved impulse control behavior due to having used the manual was reported by 73.7% in the HRT condition, 59.1% in the DC condition and 84% in the DC-is condition, which did not significantly differ across conditions. While 90.9% of the DC sample found the manual useful, this was significantly lower than in the DC-is group (95.2%). Satisfaction with the help received was significantly lower in the DC compared to the HRT group (57.9% vs. 86.6%). Satisfaction with the manual in general was lower in the DC compared to the DC-is group (61.2% vs. 82.4%).

**Table 4.** Subjective Appraisal of Participants Who had At Least Read the Manual, with Means and Standard Deviations. Percentages Comprise Response Options from “A Little” to “Absolutely”.

Item	HRT (*n* = 19)	DC (*n* = 21)	DC-is (*n* = 20)	Statistics
I think the retraining manual is good for self-help and self-guidance.	3.05 (0.91) [94.4%]	2.82 (0.66) [90.9%]	3.27 (0.70) [96.8%]	*F* (2, 60) = 1.92, *p* = .148; η_p_^2^ = .062
My impulse control disorder (e.g., nail biting, trichotillomania) decreased because of the application of the program.	2.00 (1.00) [63.2%]	1.77 (0.92) [50.0%]	2.32 (1.13) [72.7%]	*F* (2, 60) = 1.58, *p* = .214; η_p_^2^ = .050
I think the content of the manual was comprehensible.	3.68 (0.58) [100%]	3.55 (0.74) [95.5%]	3.59 (0.50) [100%]	*F* (2, 60) = 0.26, *p* = .769; η_p_^2^ = .009
I think the manual was useful.	3.05 (0.91) [94.7%]	2.55 (0.86) [90.9%]	3.23 (0.81) [95.2%]	*F* (2, 60) = 3.71, *p* = .030; η_p_^2^ = .110 (*p* = .01: DC vs. DC-is)
I was able to use the manual on a regular basis during the past weeks.	2.16 (0.96) [68.4%]	1.86 (0.89) [59.1%]	2.27 (0.93) [77.3%]	*F* (2, 60) = 1.13, *p* = .328; η_p_^2^ = .036
I had to force myself to use the manual.	2.26 (1.10) [68.4%]	2.59 (1.01) [81.8%]	2.77 (1.02) [81.8%]	*F* (2, 60) = 1.24, *p* = .296; η_p_^2^ = .040
I think the manual would make more sense if it were used in combination with psychotherapy.	2.95 (1.08) [84.2%]	2.50 (0.80) [90.9%]	3.00 (0.93) [90.9%]	*F* (2, 60) = 1.87, *p* = .163; η_p_^2^ = .059
The manual is not applicable to my impulse control disorder.	1.42 (0.77) [31.6%]	1.95 (1.00) [59.1%]	1.55 (0.80) [40.9%]	*F* (2, 60) = 2.18, *p* = .122; η_p_^2^ = .068
Due to the manual I have executed less impulse control behavior (e.g., nail biting, trichotillomania).	2.37 (1.12) [73.7%]	1.95 (1.00) [59.1%]	2.30 (1.05) [84.0%]	*F* (2, 60) =2.06, *p* = .137; η_p_^2^ = .064

*Note*. 1 = not at all, 2 = a little, 3 = a lot, 4 = absolutely. DC = decoupling; DC-is = decoupling in sensu; HRT = habit reversal training.

**Table 5.** Subjective Appraisal by Users of the Manuals (Adapted from the ZUF-8), with Means and Standard Deviations.

Item	HRT (*n* = 17)	DC (*n* = 21)	DC-is (*n* = 20)	Statistics (post hoc comparisons in brackets
How do you rate the quality of the manual? (excellent [1], good [2] vs. less good [3], not good [4])	1.94 (0.66) [94.1%]	2.10 (0.44) [85.8%]	1.95 (0.51) [90%]	*F* (2, 55) = 0.52, *p* = .595; η_p_^2^ = .019
Did you receive the type of treatment you expected to receive? (not at all [1], not really [2] vs. in general yes [3], yes absolutely [4])	3.13 (0.81) [87.6%]	2.60 (0.75) [55%]	3.11 (0.68) [83.4%]	*F* (2, 51) = 3.04, *p* = .057; η_p_^2^ = .106
To which extent did the manual met your needs? (it met all of my needs [1], it met most of my needs [2] vs. it met few of my needs [3], it did not meet my needs [4])	1.93 (0.70) [93%]	2.50 (0.95) [60%]	1.88 (0.70) [82.3%]	*F* (2, 49) = 3.37, *p* = .043; η_p_^2^ = .121
Would you recommend the manual to a friend with similar symptoms? (definitely not [1], probably not [2] vs. probably yes [3], absolutely [4])	3.29 (0.85) [88.3%]	2.86 (0.96) [66.7%]	3.30 (0.66) [90%]	*F* (2, 55) = 1.86, *p* = .165; η_p_^2^ = .063
How happy are you about the extent of the help you have received through using the manual? (dissatisfied [1], somewhat dissatisfied [2] vs. mostly satisfied [3], very satisfied [4])	3.20 (0.68) [86.6%]	2.53 (0.95) [57.9%]	3.06 (0.66) [82.3%]	*F* (2, 48) = 3.79, *p* = .030; η_p_^2^ = .136 (*p* = .022: DC vs. HRT)
Did the manual help you cope with your problems more successfully? (yes, it helped me absolutely [1], yes, it helped me a little [2] vs. no, it did not help me that much [3], no, it did not help me at all [4])	1.86 (0.53) [92.8%]	2.39 (0.70) [50%]	2.11 (0.68) [72.2%]	*F* (2, 47) = 2.67, *p* = .079; η_p_^2^ = .102
How satisfied are you with the manual in general? (very satisfied [1], mostly satisfied [2] vs. somewhat unsatisfied [3], unsatisfied [4])	1.93 (0.59) [88.7%]	2.44 (0.78) [61.2%]	2.11 (0.68) [82.4%]	*F* (2, 47) = 3.78, *p* = .030; η_p_^2^ = .138 (*p* = .021: DC vs. DC-is; *p* = .0.46: DC vs. HRT)
Would you use the manual again? (definitely not [1], probably not [2] vs. probably yes [3], yes [4])	3.40 (0.63) [93.4%]	2.95 (0.97) [73.7%]	3.44 (0.70) [88.9%]	*F* (2, 49) = 2.18, *p* = .124; η_p_^2^ = .082

*Note*. DC = decoupling; DC-is = decoupling in sensu; HRT = habit reversal training.

### Moderation

We compared all treatments against each other and entered the variables presented in Table 1 as possible moderators for treatment outcome (pre-post difference scores of the GBS) using Hayes model 1 (default settings with 5,000 bootstrap samples and standardized scores; Hayes, 2013). None of the interactions reached significance. For exploratory purposes we would like to note that the interaction of DC against the other conditions (DC-is and HRT) with lifetime skin picking achieved a (non-significant) statistical trend (coefficient: 3.66; *SE* = 2.04, *t* = 1.79, *p* = .078); DC was somewhat better for participants without a lifetime history of skin picking.

## Discussion

This is the first study to directly compare three self-help techniques (habit reversal training, decoupling and decoupling in sensu) in a heterogeneous sample with BFRBs. Decoupling in sensu and habit reversal training achieved higher subjective appraisal by participants than those in the DC group, whereas increased objective symptom improvement was observed in the DC relative to the HRT group at a (non-significant) statistical trend. A potential reason for the slight discrepancy between the subjective and objective efficacy of DC may be due to the length of the manual (15 pages relative to eight pages for HRT and DC-is). Moderation analyses revealed that DC may show the best results for patients who do not engage in skin picking, which is in line with previous results (Moritz et al., 2012).

There is a paucity of research investigating these and other self-help variants of habit reversal training. This study thus contributes preliminary results for the efficacy of such interventions in the treatment of BFRB. We indeed observed some benefits of the three techniques, especially for decoupling in sensu, which is reassuring considering that there are currently no medications approved by the FDA to treat this disorder.

As there is no gold-standard self-report measure that assesses concurrent BFRBs, another strength of our study was the introduction of the new Generic Body-Focused Repetitive Behavior Scale. The GBS is derived from other validated assessments (SPS-R: characteristics and response options; Y-BOCS: rationale to pool observations for different symptoms) and aims to capture the severity and impairment associated with different forms of BFRBs.

Several study limitations must be acknowledged. First, no long-term follow-up was carried out. Therefore, we do not know whether improvements were sustained or if some participants may show delayed changes in behavior. Second, given this study is the first to use the GBS, its psychometric properties await to be independently established; the present study suggests satisfactory internal consistency and test-retest reliability. Third, some participants commented at post-assessment that they encountered problems adapting the respective technique to their specific problem(s). Fourth, except for DC-is, adherence was rather low, with less than 60% completion for DC and HRT. Fifth, feedback was not provided for the awareness training, which is integrated in most (HRT) protocols. In addition, while competing response training is most common with static behaviors involving antagonistic muscles, some protocols recommend responses physically compatible to the target response such as playing with clay, shelling peanuts, tying and retying one’s shoes or squeezing a stress ball (Franklin & Tolin, 2007; Sharenow et al., 1989; Woods et al., 1999). Further, shorter durations of competing response training (less than 1–3 minutes) have shown efficacy as well (Twohig & Woods, 2001). It is worth noting, however, that the developers of HRT have also used a similar approach (Azrin et al., 1982). Finally, participants were recruited in part through the first author’s website, the main developer of decoupling, which may have biased results in favor of decoupling.

Given the preliminary nature of this study, several important questions and future considerations remain. First, future studies should explore whether adherence is generally lower in BFRB relative to other disorders or whether this is related more to acceptance, for example, dissatisfaction with the achieved results. It is possible that a financial incentive may raise adherence. We also suspect that some participants who had created emails for the sole purpose of study participation never checked their messages. Therefore, asking participants to automatically forward messages to their primary email address may raise completion rates. Second, long-term outcome currently remains elusive and thus future work should implement a wait-list control to examine whether the dysfunctional behavior improves or worsens in the course of an intervention trial if no (self-help) treatment is offered. Third, while a subgroup of participants reported substantial benefits, this subgroup still comprised the minority. Interestingly, more than 80% of participants confirmed that the intervention would make more sense if it were used in combination with psychotherapy. It would thus be interesting to explore whether the efficacy of the techniques may be augmented upon direct administration by a therapist or via other media (e.g., video). Indeed, it is also possible that the effect of HRT was underestimated given that this technique is usually delivered by a therapist and given that we included only two of its elements, which however represent the core of HRT (Miltenberger et al., 1985). Fourth, whether the concurrent application of the different techniques yields surplus effects or leads to interference (thus compromising efficacy) also requires examination. Finally, we suspect that the decoupling techniques would be more effective for those presenting with automatic behaviors, while HRT is likely better when the behavior is focused, which also remains to be tested.

To conclude, this study is a necessary first step toward demonstrating the preliminary efficacy of three brief self-help interventions to treat BFRB. All techniques are available for both affected persons and clinicians at no cost (www.uke.de/decoupling). Since positive treatment outcomes were observed in only a subgroup of participants, further refinement and ongoing examination is required to increase treatment efficacy for BFRBs.
